# Immunotherapy and the Spectrum of Kidney Disease: Should We Individualize the Treatment?

**DOI:** 10.3389/fmed.2022.906565

**Published:** 2022-06-14

**Authors:** Sheila Bermejo, Mónica Bolufer, Mar Riveiro-Barciela, Maria José Soler

**Affiliations:** ^1^Nephrology Department, Vall d'Hebron Hospital, Barcelona, Spain; ^2^Centro de Referencia en Enfermedad Glomerular Compleja del Sistema Nacional de Salud (CSUR), Barcelona, Spain; ^3^Liver Unit, Internal Medicine Department, Hospital Universitari Vall d'Hebron, Barcelona, Spain; ^4^Centro de Investigación Biomédica en Red de Enfermedades Hepáticas y Digestivas (CIBERehd), Instituto de Salud Carlos III, Madrid, Spain; ^5^Department of Medicine, Universitat Autònoma de Barcelona (UAB), Barcelona, Spain

**Keywords:** dialysis, chronic kidney disease, renal transplant, immunotherapy, renal biopsy

## Abstract

The new targeted cancer therapies including immune checkpoint inhibitors (ICIs) have been demonstrated to improve the survival of oncological patients, even in cases of metastatic cancer. In the past 5 years, several studies have revealed that ICI can produce several immune-mediated toxicities involving different organs, such as the skin, the gastrointestinal tract, the liver, and, of course, the kidney. The most frequent lesion of immunotoxicity in the kidney is acute interstitial nephritis (AIN), although other nephropathies have also been described as a consequence of the use of ICI, such as glomerulonephritis and acute thrombotic microangiopathy, among others. In addition, kidney rejection has also been reported in kidney transplant patients treated with ICI. Normally randomized clinical trials with ICI exclude patients with end-stage kidney disease, namely, patients undergoing dialysis and kidney transplant patients. Several important questions need to be addressed in relation to immunotherapy and patients with kidney disease: (a) when to start corticosteroid therapy in a patient with suspected acute kidney injury (AKI) related to ICI, (b) the moment of nephrologist referral and kidney biopsy indication, (c) management of ICI in patients undergoing dialysis, and (d) the effect of ICI in kidney transplantation, immunosuppressive personalized treatment, and risk of allograft rejection in kidney transplant patients. The objective of this review was to summarize the recently published literature on a wide spectrum of kidney disease patients with cancer and ICI. This review will address three main important groups of individuals with kidney disease and cancer immunotherapy, AKI associated with ICI, patients undergoing dialysis, and kidney transplant recipients. We believe that the information provided in this review will enlighten the personalized ICI treatment in individuals with a broader spectrum of kidney diseases.

## Introduction

Cancer is an important cause of death worldwide and is expected to be the first cause of death in many countries in years to come since stroke and heart disease show a decrease in mortality (1). According to the data provided by the World Health Organization (WHO) in 2019, cancer was the first or second cause of death in subjects below 70 years in 112 out of 183 countries, and it ranks third or fourth in other 23 countries (2). Thus, in 2020, 19.3 million new cases of cancer were diagnosed and 10 million deaths were recorded worldwide (1). The increase in the incidence and mortality of cancer has been mainly related to aging of the population and the increase in risk factors for the development of cancer (1). Given the important incidence of cancer, the scientific community has made efforts in recent years to develop new therapies for these patients. One of the emerging therapies is under the premise of stimulating the patient's own immune system to deal with cancer cells: therapies based on vaccines, oncolytic viruses, T cell-directed therapies, bi-specific antibodies, and checkpoint inhibitors (3). With the use of these new therapies, an increase in patient survival has been evidenced (4). Thus, the tumor microenvironment is composed of various escape routes from the recognition of the immune system, which allows the growth and dissemination of cancer cells, causing metastasis. An important mechanism is the expression of inhibitory ligands for CTLA-4 and PD-1 receptors on T cells and other immune cells that cause inhibition of the tumor microenvironment, known as immune checkpoints that deactivate T cells (5). Here remains the pathophysiological mechanism of the use of immune checkpoint inhibitors (ICIs), which bind to CTLA-4 and PD-1 to activate immune cells from a quiescent state to cause a reaction against tumor cells (3). However, this mechanism is not selective, and the use of ICI increases the incidence of immune-related adverse events (irAEs). The gastrointestinal tract, the skin, the endocrine system, and the liver are the most frequent locations where irAEs occur, with an incidence that ranges between 15 and 90% (6, 7). The kidney may also be involved in the damage caused by irAEs with an estimated incidence of 3–5% (8–10). The kidney pathology most frequently associated with the use of ICI is acute interstitial nephritis (AIN), although cases of glomerulopathies and thrombotic microangiopathies, among others, have also been described (11). In patients with acute kidney injury (AKI) associated with ICI, it is crucial to know when to perform a kidney biopsy and to start treatment, since it has been shown that the time of starting treatment is important for kidney prognosis (12). As the use of ICI has demonstrated impressive results in patients with advanced cancer, their use has been increasing in recent years including people with a kidney transplant or with a chronic kidney disease grade 5 treated by dialysis (CKD5D). In kidney transplant patients, the use of ICI has been associated with an increased risk of acute rejection, making it necessary to individualize immunosuppressive therapy and close monitoring, especially if concomitant to kidney replacement therapy (KRT) (13). In addition, the use of ICI has also been extended to the population with CKD5D, including both hemodialysis (HD) and peritoneal dialysis (PD) therapy. However, in these two scenarios, experience is limited and the literature is scarce (14). Altogether, the spectrum of kidney disease is wide around the use of ICI, and different clinical situations of patients with cancer and kidney disease must be considered. The intention of this review is to address the entire spectrum of all kidney patients receiving ICI. For that purpose, the kidney complications derived from the treatment and its use in the renal population such as renal transplant patients and patients with CKD5D receiving KRT in the form of HD and PD will be addressed.

## Native Kidney Injury Associated With Immunotherapy

As mentioned above, AIN is the most frequent (80–93%) histopathological lesion documented associated with ICIs in patients with acute kidney failure (8, 11, 15–19). Gupta et al. published a multicenter study enrolling a huge cohort of patients with AKI associated with immunotherapy: a total of 429 patients with AKI associated with a checkpoint inhibitor (ICI-AKI) were compared with 429 controls who received the same treatment but they did not develop any kidney complications (12). In this study, a total of 125 kidney biopsies (82.7%) were diagnosed with AIN, with a latency time of 16 weeks (8–32 weeks) before the start of the treatment with ICIs and AKI; however, the AKI episode occurred in the first year after starting treatment only in 11.4% of cases. It is important to take into account the presence of hematuria in almost 40%, pyuria in more than 50%, and proteinuria and increased blood eosinophils in 16.5% of patients. Several studies focused on assessing the risk factors for developing AIN. The following characteristics have been identified as risk factors for ICI-AKI: (1) the association with other drugs, which happens between 60 and 75% of cases (8, 12, 15), mainly proton pump inhibitors; (2) lower baseline estimated glomerular filtration rate (eGFR) (8, 12); (3) combined ICI therapy (8); (4) arterial hypertension (17); (5) prior or concomitant extrarenal irAEs such as rash and hepatitis as the most common (12, 17). Concomitant treatment with other drugs that increased the risk to develop AIN and the early start of corticosteroids in <2 weeks are the most important factors for recovering kidney function in these patients (8, 12). In contrast, stage III of AKIN, lung cancer, and concomitant irAEs are risk factors for nonrecovery kidney function (8). In a recently published study by Garcia-Carro et al., the following were identified as risk factors for mortality: the type of cancer (not melanoma, lung, or urogenital malignance), the type of ICI, and the presence of an episode of AKI (20).

Glomerular diseases have also been described in patients with AKI associated with ICI treatment (Table 1) (8, 11, 12, 21–25). A large series of kidney biopsies was described in around 3–8% of the cases in some studies (12, 15) and in up to 41% of cases in another study, with a concomitant association of glomerulopathy and AIN (26). The glomerular pathology associated with ICI can be classified based on the clinical presentation: 1. the nephrotic syndrome as a clinical presentation of podocytopathies such as minimal change diseases or focal segmental glomerulosclerosis, amyloidosis, and membranous nephropathy and 2. the nephritic syndrome as a clinical presentation of pauci-immune vasculitis (11), complement 3 glomerulonephritis (G3GN) (24), immunoglobulin A nephropathy (27), IgA dominant postinfectious glomerulonephritis (23), anti-glomerular membrane disease, thrombotic macroangiopathy, immune complex glomerulonephritis, and lupus-like nephritis (25). The most frequent pathologies described are pauci-immune vasculitis (26.7%), podocytopathies (24%), and C3GN (11.1%) (26). The majority of these patients received corticosteroids (98%), and immunotherapy was discontinued (88%).

**Table 1:** Glomerulopathies and ICIs.

**Case**	**Renal manifestation**	**Therapy**	**Response**
**Clinical features of patients receiving anti-PD-1**			
Daanen (21)	Nephrotic syndrome FSGS	DI + Steroids + MMF	Remission flowed by proteinuria relapsed
Kitchlu (22)	Nephrotic syndrome MCD	DI + Steroids	Partial remission
Mamlouk (11)	Membranous nephropaty IgA. Non proliferative lesions Focal necrotizing pauci-immune glomerulonephritis no crescents	DI + Steroids DI + Steroids + MMF + Infliximab DI + Steroids + plasmapheresis + Rituximab	Remission Partial remission Partial remission
Jung (23)	IgA dominant postinfectious glomerulonephritis	DI + Steroids + RRT	Remission
Cortazar (8)	Pauci-immune GN ANCA negative	Steroids + Rituximab	Remission
Ashour (24)	Diffuse endocapillary proliferative GN with cellular crescents Complement 3 glomerulonephritis	DI + Steroids	Partial remission
Gupta (12)	AA Amyloidosis Membranous with lupus-like features	Tocilizumab IVIG	No recovery No recovery
**Clinical features of patients receiving anti-CTLA4**			
Mamlouk (11)	Nephrotic syndrome. Endocapillary hypercellularity	DI + Steroids	Remission followed by relapsed
Gupta (12)	Pauci-immune GN	DI+Plasmapheresis + Rituximab	No recovery
**Clinical features of patients receiving Anti CTLA4** **+** **Anti PD-1**			
Kitchlu (22)	MCD	DI + Steroids	Remission
Mamlouk (11)	Focal segmental pauci-immune glomerulonephritis with no cresents MPO + ANCA	DI + Steroids + Plasmapheresis + Rituximab	Partial remission
Fadel (25)	Extramembranous and mesangial deposits (IgG, IgM, C3 and C1q) and + ds DNA	DI	Partial remission
**Clinical features of patients receiving Anti PD-L1**			
Gupta (12)	Pauci-immune GN	Rituximab	No recovery

*ICIs, immune checkpoint inhibitors; FSGS, focal segmental glomerulosclerosis; DI, discontinuation immunotherapy; MMF, mycophenolate mofetil; MCD, minimal changes disease; RRT, renal replacement therapy; IVIG: intravenous immunoglobulin*.

## When to Start Corticosteroid Therapy in a Patient With Suspected AKI Related to ICI?

A few clinical guidelines have focused on the diagnostic and therapeutic management of patients with AKI secondary to the use of ICI (28–30) (Table 2). Due to the lack of studies on this topic or randomized clinical trials that evaluate the use of corticosteroids by comparing different doses and timings, our conclusion must be considered cautiously due to several potential limitations in the available data. To the best of our knowledge, currently, there are no randomized clinical trials for answering the proposed questions, and for that reason, the level of evidence for recommending when to start or tapering steroids in these patients is only based on published daily clinical practice and guidelines. Kidney damage can occur with a decline in kidney function and/or the presence of proteinuria. If proteinuria is <1 g, the recommendation is to continue with the same dose of ICI and monitor and follow up (30). If proteinuria is 1–3.5 g/24 h, kidney biopsy should be considered, especially in cases of persistent proteinuria or progressive increase, and ICI therapy should be stopped until histological confirmation of a possible glomerulopathy. Once diagnosed, glomerulopathy treatment and the possibility of ICI reintroduction will be based on physiopathology. In the case of acute kidney function decline, the current guidelines recommend the clinical decision depending on the level of deterioration: if creatinine increases between 1 and 1.5 times the basal level, ICI should be stopped, dehydration corrected, and all potential nephrotoxic drugs should be avoided. Kidney function monitoring should be performed between 3 and 7 days (28–30). If the increase in creatinine is between 1.5 and 3 times the baseline level, the ICI should be stopped (28–30). The start of corticosteroids at a dose of 0.5–1 mg/kg is also recommended. If the deterioration is more severe, such as an increase of more than 3 times the basal level, the ICI should be definitively stopped and corticosteroid therapy should be started at a dose of 1 or 2 mg/kg. In the cases that do not respond to corticosteroid treatment, another immunosuppression therapy should be assessed (28). If the deterioration is even greater with an increase of more than 6 times the baseline value or need for KRT, intravenous corticosteroid pulses should be started followed by oral prednisone at 1–2 mg/kg. The use of other immunosuppressants should be considered if improvement has not been observed after 1 week of corticosteroids (28–30).

**Table 2:** Recommendations of clinical guidelines (NCCN Guidelines for Management of Immunotherapy-Related Toxicities and American Society of Clinical Oncology (ASCO) guidelines) (33, 34) in AKI in patients treated with immunotherapy.

**Clinical conditions**	**Management**	**Treatment**
**Mild cases**		
sCr 1–1.5 x baseline	Withhold ICI Monitor renal function every 3–7 days	Correct dehydration, Withdraw nephrotoxic medication
Proteinuria <1 gr/24 h	Continue ICI	Monitoring
**Moderate cases**		
sCr 1.5–3 x baseline	Withhold ICI Monitor renal function every 3–7 days	Nephrology consultation +/- start corticotherapy (0.5–1 mg/Kg/24 h)
Proteinuria 1–3.5 gr/24 h	Consider kidney biopsy Withhold ICI if kidney biopsy confirms	Treat the renal pathology diagnosed
**Severe cases**		
sCr >3 x baseline or > 4 mg/dl	Kidney biopsy Permanent discontinuation of ICIs	Start corticosteroid therapy (1–2 mg/Kg/24 h)
Proteinuria >3.5 gr/24 h	Kidney biopsy Withhold ICI if kidney biopsy confirms	Treat the renal pathology diagnosed
**Life-threating cases**		
sCr > 6 x baseline or dialysis indicated	Kidney biopsy Permanent discontinuation of ICIs	Intravenous bolus corticosteroid If no response, consider other immunosuppressive agents (MMF, CTX, AZA or infliximab)

*AKI, acute kidney injury; MMF, mycophenolate mofetil; CTX, cyclophosphamide; AZA, azathioprine*.

## When Should the Patient With AKI and ICI be Referred to a Nephrologist? Biopsy or No Biopsy AKI in Patients With ICI?

Cancer patients with AKI will benefit from the assessment of a specialist in nephrology who will evaluate the risks and benefits of performing a kidney biopsy (31). There is no scientific evidence regarding the moment of AKI related to ICI referral to a nephrologist, and our suggestions are mainly based on the published guidelines. In brief, if the increase in creatinine is more than 1.5 times the baseline level, consulting a nephrologist is recommended for assessing the need for a kidney biopsy.

One of the important decisions in these patients is when nephrologists should indicate a kidney biopsy, the “gold standard” for kidney disease diagnosis and prognosis. In patients with cancer undergoing treatment with immunotherapy, it is important to identify those who present AKI secondary to acute tubular necrosis with the purpose of avoiding unnecessary treatment with corticosteroids and the temporary discontinuation of immunotherapy. Furthermore, the accurate diagnosis of both interstitial and glomerular kidney pathology will have treatment and prognostic implications.

At present and based on expert opinion and the American Society of Clinical Oncology (ASCO) guidelines (29), kidney biopsy in patients undergoing ICI treatment should be performed if there is proteinuria > 3 g, oliguria, dysmorphic hematuria, and suboptimal response to empirical treatment with corticosteroids (22, 32). However, according to recently published studies, kidney biopsy should be strongly considered if there are several alternatives that justify acute kidney failure (33). In several cases, it is difficult to differentiate AIN from acute tubular necrosis. However, novel urinary cytokine biomarkers that would help to differentiate among them, such as IL-9 and TNF-alpha, are currently under development (34).

However, in cases with severe AKI secondary to ICI with advanced palliative cancer, a kidney biopsy is not mandatory to start corticosteroid therapy. For that reason, strategies for developing biomarkers of AKI associated with ICI may be useful individualizing treatment and diagnosis in the future.

## Management of ICIs in Patients Undergoing Dialysis

A high incidence of several types of cancer has been identified in patients undergoing dialysis (35). Additionally, these patients are normally excluded from most clinical trials with cancer therapies, since most of them are aimed to study the pharmacodynamic and pharmacokinetic characteristics of these drugs. In the case of ICIs, these are not modified by the use of dialysis due to their molecular size (36) and do not require dose adjustment. Thus, theoretically, the use of ICI in dialysis patients seems to be safe, although the literature on this topic is scarce (37). Cancer that is most associated with the use of ICI in patients undergoing dialysis is renal carcinoma, followed by genitourinary and melanoma. Nivolumab and pembrolizumab are the two most commonly used drugs (36). Since this population is excluded from clinical trials, evaluating safety in patients undergoing dialysis is a challenge (38). In the previously reported case series, the majority of the adverse events are grades 1 and 2, and the most common adverse effect is hematological, followed by skin and gastrointestinal involvement. A higher frequency of hematological adverse effects has been showed in patients undergoing dialysis than in the general population (39), but the rest of the toxicities have been evidenced less frequently. Published studies that included more than 5 dialysis patients under treatment with ICI, i.e., type of cancer, type of ICI, and outcomes, are summarized in Table 3 (40–45). As expected, the risk of developing irAEs in patients undergoing dialysis seems similar to the general population. A plausible explanation for this is that the excretion of ICIs is not renal, so it is logical that the frequency of adverse reactions is similar in both populations. Management of these immune-mediated toxicities is the same, based on the use of corticosteroids. However, patients undergoing dialysis have many comorbidities, and for that reason, the exposure to corticosteroid therapy must be limited to avoid its adverse effects (46). It is important to highlight that, in dialysis patients who are recipients of a previous kidney transplant, the use of ICI may lead to allograft rejection. The use of mini-pulse steroids can be considered during the first weeks of starting treatment with ICI to prevent allograft intolerance (13, 47). It is worthy to mention that the mortality of dialysis patients with cancer is very high compared with the general population (48). In addition, the incidence of cancer in patients undergoing dialysis is 9.5% higher than in the general population (35, 49). Survival and cancer prognosis in patients undergoing dialysis and immunotherapy is not well known. Therefore, more studies are needed to evaluate the tolerance and the incidence of irAEs derived from ICIs in this population.

**Table 3:** The spectrum of the use of ICI in patients undergoing dialysis.

**Studies**	**Year**	**Number of patients**	**More frequent type of cancer**	**More used type of ICI**	**Adverse events**
Strohbehn et al. (40)	2020	19	Genitourinary	Pembrolizumab and nivolumab	Grades 3–4 myocarditis and pneumonitis
Kuo et al. (41)	2020	11	Urothelial	Pembrolizumab	Grade 3 and 4 anemia
Vitale et al. (42)	2019	8	Renal cell carcinoma	Nivolumab	Grade 3 diarrhea, asthenia and anorexia
Hirsch et al. (43)	2020	8	Urothelial	Pembrolizumab	Dermatitis
Jain et al. (44)	2020	8	Melanoma	Pembrolizumab	Pneumonitis
Tachibana et al. (45)	2019	7	Renal cell carcinoma	Nivolumab	Grade 3 fatigue

*ICI, immune checkpoint inhibitor*.

## ICIs in Kidney Transplantation

**Figure 1:**
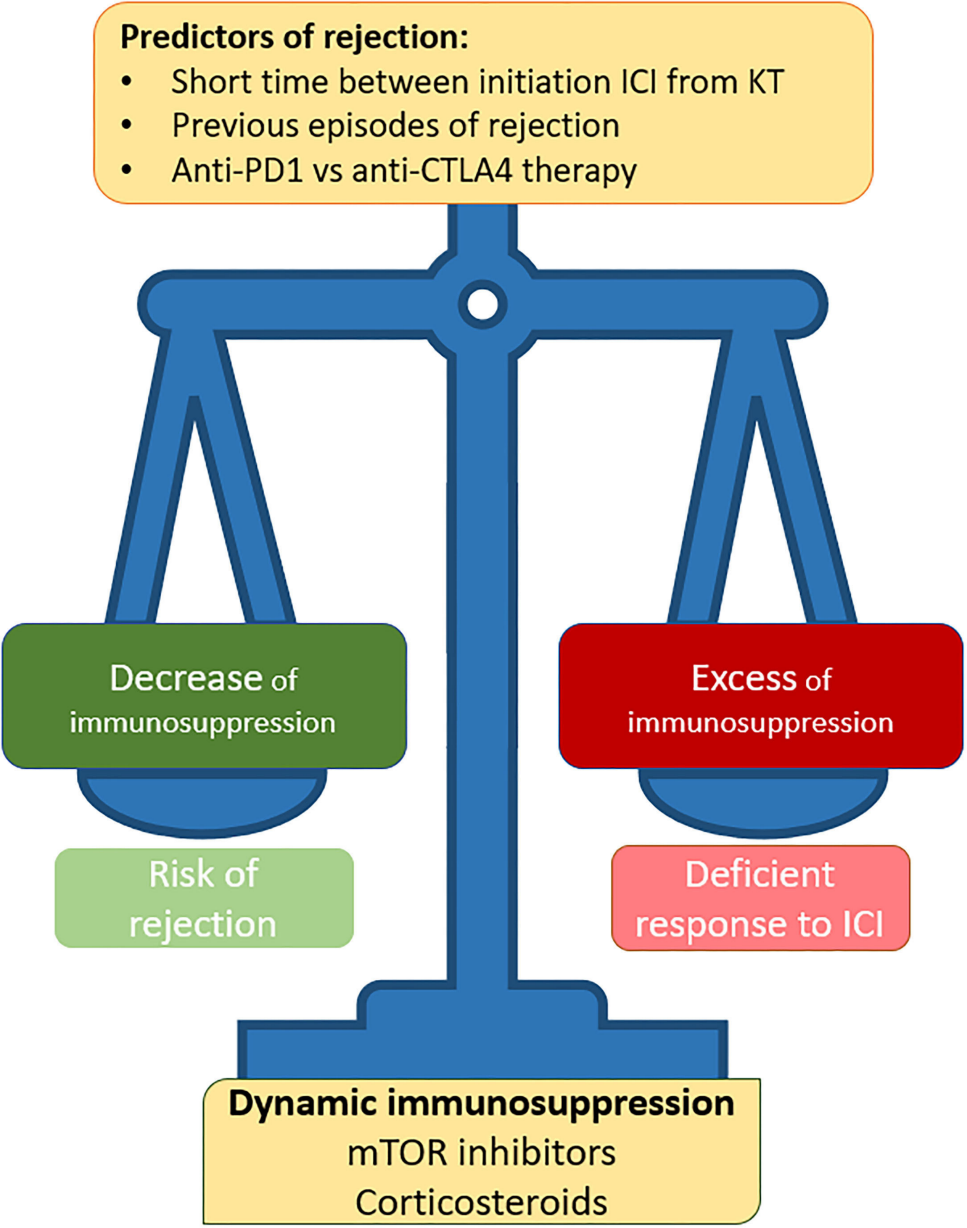
Controversies over the use of ICI therapy in kidney transplant patients.

Kidney transplant patients have a 3-fold risk of developing cancer than the general population (50), and it is the second cause of death in this population (51–53). The survival of kidney transplant recipients with cancer is lower than the general population (52). Skin tumors are the most common type of cancer in these patients (54), the use of ICI is highly relevant in this type of cancer, and its treatment with the new targeted therapy has been revolutionized in the past decade (14). Unfortunately, as happens with dialysis patients, kidney graft recipients were usually excluded from most clinical trials with ICIs, and for that reason, there is scarce literature regarding the use of ICIs in this setting. The use of ICI is a challenge in kidney transplant patients for the following two reasons: (1) the use of ICI increases the risk of presenting acute rejection related to the activation of T-type cellular immunity and (2) the use of immunosuppressants can compromise the antitumor activity of immunotherapy (55–57) (Figure 1). Thus, it is crucial to individualize the type of ICI used and the immunosuppressive therapy in each case. The risk of rejection increases if the use of ICI is closer to the kidney transplant intervention (55). Anti-CTLA-4 agents appear to have a trend toward a lower risk of rejection compared with anti-PD-1/PD-L1 therapies (58). This may be related to the fact that CTLA-4 plays a fundamental role in the activation of the immune response in the lymph nodes, which has been less frequently associated with rejection; instead, PD-1 and PD-L1 have a key role in the immune activation in the peripheral system (59). In a recently published series, a 40–50% incidence of acute rejection has been described with the use of ICI in transplant patients (13, 14). Usually, the type of rejection observed is the cellular type without the development of donor-specific antibodies (60); however, Murakami et al. reported in their series of kidney transplant patients with ICI (*n* = 69) that 50% of the rejections were T cell-mediated rejection and the rest were mixed (T cell-mediated and antibody-mediated rejection) (13). The onset of rejection is relatively close to the start of ICI treatment, with a median of 22–24 days (13, 61). In the transplant setting, it is important to differentiate the appearance of rejection and AIN. AIN more frequently presents eosinophilic nodules and an absence of arteritis (8). In addition, the timing from ICI initiation to the development of the renal event differs, with rejection occurring earlier, whereas AIN is usually a later adverse event (61).

Regarding immunosuppression, in kidney transplant patients with cancer, management with mTOR inhibitors is recommended. In addition, it has been shown that the use of mTOR in transplanted patients under treatment with ICI seems to reduce the risk of rejection (13). In addition, patients receiving single-agent prednisone (≤ 10 mg/day) at CPI initiation seemed to have numerically higher tumor responses to CPI therapy than those receiving single-agent mTOR inhibitors, calcineurin, or combination immunosuppressant therapy regimens (56). The use of “dynamic immunosuppression” has also been described to reduce the risk of acute rejection (47, 62), although the efficacy of this regimen in an ongoing prospective study has yet to be demonstrated (NCT 04339062). Cancer prognosis and overall survival in metastatic diseases have been shown to have better survival in the kidney transplant population treated with ICI than in those who do not receive ICI (13). In melanoma, it has been shown that the use of anti-PD1 in monotherapy in the kidney transplant population has less efficacy than in the general population (63, 64). However, when the combination of anti-PD1 and anti-CTLA-4 is used, response rates are the same as those in the non-transplanted population (65). Taking all together, the use of ICI in kidney transplant patients is an opportunity to improve cancer prognosis in kidney transplant patients; however, individualized management is necessary in terms of immunosuppression, and each case must be approached from a multidisciplinary point of view.

## Biomarkers of ICI-Induced AIN and Kidney Allograft Rejection

The underlying mechanisms of ICI-AIN are unknown; however, some hypotheses have been postulated as follows: (1) the presence of T cells are reactive against autoantigens expressed in the kidney (66); (2) generation of anti-kidney antibodies (25, 67); (3) cytokine-mediated injury secondary to T-cell activation (68); (4) possibility of preexisting subclinical autoimmune disease (69) and finally (5) loss of tolerance of T cells that had been previously stimulated by other drugs that also induce AIN (58, 70).

Currently, new biomarkers are being developed to early identify renal failure associated with checkpoint inhibitors and their prognoses such as IL 17, sCD163 (soluble receptor expressed from M2 macrophages), IL 6, and blood levels of lactate dehydrogenase (70). Moledina et al., in a prospective study of 218 patients where 15% were diagnosed with AIN, found that urinary levels of tumor necrosis factor-alpha (TNF-alpha) and IL-9 were higher in this group as compared with other biopsied kidney pathologies such as acute tubular necrosis, diabetic nephropathy, or glomerulopathies (34). Another biomarker that can help differentiate interstitial from glomerular pathology is the composition of macrophage subtypes in urine. While the predominance of M1 in urine suggests acute renal failure secondary to AIN, the dominance of M2 in urine could be a source of biomarkers of kidney disease progression, mainly in crescentic glomerulonephritis (71). In cancer patients with renal failure and without the possibility of renal biopsy, it may be difficult to differentiate AIN from acute tubular necrosis, and some urinary cytokines are being studied and developed to facilitate the differential diagnosis, namely, urinary I-TAC/CXCL11, CLXCL10, IL-6, and MCP-1 (72). Finally, Isik et al., in an elegant study of 37 patients where they compared ICI-AKI with non-ICI-AKI, showed that serum C-reactive protein and urine retinol-binding protein/urine creatinine (uRBP/Cr) can be plausible markers to differentiate both types of kidney failure (73).

In the case of kidney transplantation, the histopathological similarity between T cell-mediated rejection and ICI-associated AIN presents a clinical challenge. Recently, interferon alpha-inducible protein 27 (IFI27) gene expression in kidney tissue has been identified as a potential marker to differentiate between both entities (74).

## Conclusion

The ICI spectrum in kidney disease is wide, from its related immunotoxicity such as AIN and glomerulonephritis to their use in special populations, namely, dialysis and kidney transplant patients. In this review, we highlighted the renal irAEs associated with ICI treatment in patients with advanced cancer. In addition, we also demonstrated that there is an urgent need for randomized clinical trials with ICI involving patients with end-stage kidney disease and kidney transplant recipients. We also addressed some open questions for helping in the daily clinical practice, including when to start corticosteroid therapy in a patient with suspected AKI secondary to ICI, when to refer to the nephrologist or indicate kidney biopsy, the safety of ICI in patients undergoing dialysis, and ICI suggestions in kidney transplant patients.

## Author Contributions

SB, MB, MR-B, and MS have collaborated on the original idea. SB, MB, and MS wrote the paper. All authors approved the final version of the submitted manuscript.

## Funding

This research was funded by ISCIIII-FEDER and ISCIII-RETICS REDinREN, Grant Numbers PI17/00257, PI21/01292, RD16/0009/0030, and RICORS RD21/0005/0016. Enfermedad Glomerular Compleja del Sistema Nacional de Salud (CSUR), enfermedades glomerulares complejas.

## Conflict of Interest

SB reports honorarium for conferences, consulting fees and advisory boards from AstraZeneca and Mundipharma. MS reports honorarium for conferences, consulting fees and advisory boards from Astra Zeneca, NovoNordsik, Esteve, Vifor, Bayer, Mundipharma, Ingelheim Lilly, Jan-sen, ICU Medical, and Boehringer. The remaining authors declare that the research was conducted in the absence of any commercial or financial relationships that could be construed as a potential conflict of interest.

## Publisher's Note

All claims expressed in this article are solely those of the authors and do not necessarily represent those of their affiliated organizations, or those of the publisher, the editors and the reviewers. Any product that may be evaluated in this article, or claim that may be made by its manufacturer, is not guaranteed or endorsed by the publisher.
